# The influence of lifestyle habits on levels of depression among rural middle school students in Northeastern China

**DOI:** 10.3389/fpubh.2024.1293445

**Published:** 2024-01-29

**Authors:** JunCheng Zhao, Xiaoyin Wang, Shiliang Xu, Wenjing Yan, Jingzhe Wang, Ende Wang, Tao Liu, Ming Hao

**Affiliations:** ^1^School of Public Health and Health Management, Gannan Medical University, Ganzhou, China; ^2^College of Electronics and Information Engineering, Liaoning Institute of Science and Technology, Benxi, Liaoning, China

**Keywords:** depression, physical activity, sleep quality, screen time, household income

## Abstract

**Background:**

Depression rates among adolescents have risen dramatically over the past decade. Therefore, preventing depression among adolescents is particularly important. Differences in lifestyle habits may play a role in depression.

**Purpose:**

This study aimed to explore the influence of living habits on depression levels among rural middle school students in Northeast China and to provide a theoretical basis for developing interventions to reduce depression levels in middle school students.

**Methods:**

A total of 296 middle school students aged 13–15 years from Benxi City, Northeast China completed the anthropometric measurements, Physical Activity Scale-3 (PARS-3), and the Self-Rating Depression Scale (SDS). Their average screen time in the most recent week, parents' education level, and monthly family income were collected through a questionnaire.

**Results:**

Females had higher depression scores than males (41.0 ± 6.9 vs. 37.9 ± 8.0). Physical activity (β = −0.38, *t* = −7.06, *P* < 0.01), family income (β = −0.20, *t* = −4.07, *P* < 0.01), screen time (β = 0.16, *t* = 3.34, *P* < 0.01), age (β = 0.15, *t* = 3.16, *P* < 0.01), sex (β = −0.13, *t* = −2.74, *P* < 0.01), and sleep quality (β = −0.08, *t* = −1.87, *P* < 0.01) are important factors related to depression levels.

**Conclusion:**

The preliminary analysis results showed that among middle school students in rural Northeast China, the depression level of females was significantly higher than that of males. Poor quality sleep, low levels of physical activity, low household income, and long screen time were positively associated with depression. Therefore, strengthening physical activity, improving sleep quality, and reducing screen time are of clinical relevance in preventing and reducing depression.

## 1 Introduction

Currently, depression is the most common mental disorder worldwide (1). Between 1990 and 2018, there were 322 million cases of depression reported globally (2). It is estimated that depression will become the most burdensome disease worldwide by 2030 (3). A meta-analysis of 72 studies on the global prevalence of depression among adolescents showed that between 2010 and 2020, the prevalence of depressive symptoms among teenagers rose from 24 to 37% (4). Mental health problems are equally serious among Chinese adolescents. According to the results of China's 2022 National Depression Blue Book, children under the age of 18 years in China account for ~30.28% of the total number of people suffering from mental health problems, such as various emotional and behavioral disorders, and ~5.2% have obvious mental health problems. Depression is one of the most prevalent mental health disorders and can lead to anxiety, sleep disturbance, and other adverse outcomes. Patients with major depressive disorders may exhibit suicidal behaviors (5). Therefore, it is important to improve the depression conditions in adolescents, particularly as this is a period of rapid social, emotional, and cognitive development, as well as critical life transitions.

However, its pathogenesis remains unclear. For mild to moderate depression, there is no reliable evidence that any single treatment method has a prominent effect on improving depressive symptoms (6). Nevertheless, lifestyle habits play a crucial role in the emergence of depression (7, 8), and the importance of self-care behavior in preventing depression needs to be emphasized (9). Maintaining appropriate exercise and sufficient sleep are considered beneficial for improving and preventing depression.

Physical exercise has a positive impact on the prevention and treatment of depression (10). An exercise intervention study of 433 adolescents with depression showed that moderate- to high-intensity exercise had a significant therapeutic effect on adolescent depression and was significantly better than low-intensity exercise, in addition to the moderate and sustained positive effects of the exercise intervention on adolescent depression (11).

Sleep disturbance is also considered an important cause of depression in adolescents. Three quarters (76.5%) of the 28,135 teenagers did not get the necessary 8 h of sleep on a typical school night (12). The results of a cross-sectional study of the relationship between sleep duration and depression in rural areas of Northeast China showed that sleep duration was strongly associated with depression in a rural population in Liaoning Province. Inadequate sleep duration is a risk factor for depression. Therefore, optimizing sleep status may contribute to physical and mental health (13). Increasing use of electronic devices among students has been cited as a significant contributor to sleep disturbances and reduced average sleep duration. A study on the relationship between mobile phone addiction and the incidence of low and short sleep among 1,125 adolescent students in South Korea showed that the frequent use of mobile phones is a risk factor for sleep disorders in adolescents (14). Another study on the relationship between cell phone use and depressive symptoms, body aches and daytime sleepiness among secondary school students in Hong Kong showed that cell phone use was associated with a wide range of health problems, such as depression, sleep disorders, etc., and that secondary school students who had a higher demand for cell phones scored significantly higher in terms of depression severity and daytime sleepiness (15).

A study on the development and changes in depression during adolescence showed that middle and late adolescence (15–18 years old) is a critical period for susceptibility to depression because the incidence of depression is higher during this period (16). In a study of the impact of schooling on depressive symptoms in Chinese adolescents before the COVID-19 outbreak, it was found that the negative impact of schooling on the mental health of Chinese adolescents was even greater than that of the epidemic and home isolation, and that secondary school students were subjected to unprecedentedly high levels of pressure to advance to higher levels of schooling, which may have had a greater impact on the incidence of depression among secondary school students (17). In addition, in a study of 1,314 schoolchildren on the impact of poverty mediated by family social capital on children's anxiety and depression, it was shown that poverty had a significant direct impact on children's anxiety and depression (18).

This study aimed to investigate the status quo of depression levels of middle school students in rural areas of Northeast China, analyze the factors influencing their depression levels, explore the effects of lifestyle habits (physical activity, screen time, and sleep quality) on depression levels, and give a theoretical foundation for developing interventions to reduce the depression levels in middle school students.

## 2 Materials and methods

### 2.1 Participants

We have chosen the Rural Department of Mingshan District, Benxi City, Liaoning Province as the survey area. There are three middle schools in the local area, two of which have a total of <80 students and are no longer continuing to recruit new students. We selected the remaining middle school, which had the largest number of students among the three rural middle schools in the district. Schools in this area were suspended for ~1 month because of the COVID-19 pandemic in early 2020. No cluster epidemics occurred before September 2021, and the students attended classes normally. We held parent teacher meetings and required parents and children to sign informed consent forms and fill out survey questionnaires on site. In total, 318 students agreed to participate in the questionnaire survey, while parents provided guidance. Of the participants, 296 students (male: 158; female: 138) provided complete and valid data to be included in this study.

### 2.2 Physical activity score

The physical activity levels of rural children were investigated using the Physical Activity Scale-3 (PARS-3) (19). The scale consists of three questions examining the intensity, duration, and frequency of exercise. A five-level scoring method of 1–5 points was used for evaluation. The exercise intensity, time, and frequency were multiplied to determine the amount of exercise. Exercise volume ranged from 0 to 100 points, with higher scores indicating greater levels of exercise. Categories were defined as: light exercise: 0–19 points; moderate exercise: 20–42 points; and strenuous exercise: 43–100 points (19).

### 2.3 Screen time

Through a questionnaire, the students were asked to record their average screen time usage (min/day) in the most recent week. To estimate screen time, they were asked to separately report the time spent watching TV, using a computer, and playing video games.

### 2.4 Sleep quality score

We use the Pittsburgh Sleep Quality Index (PSQI) to evaluate the sleep status of students. The PSQI was compiled by Dr. Buysse, a psychiatrist at the University of Pittsburgh, in 1989 (20). This scale consists of seven components (sleep latency, duration, and disturbance, daytime dysfunction, habitual sleep efficiency, sleep medication, and overall sleep quality) and a total of 19 questions. The sum of the scores for the seven components is the PSQI score, and the higher the score, the worse the sleep quality (20).

### 2.5 Depression score

Depressive symptoms were assessed using the Self-Rating Depression Scale (SDS) (21), which is a self-reported measure of depression consisting of 20 items covering the emotional, psychological, and physical characteristics of depression. Each item is ranked from 1 to 4, with higher scores indicating greater levels of depressive symptoms, resulting in a total score of between 20 and 80. All participants were then divided into two groups: (1) a non-depressive symptom group (SDS < 40) and (2) a depressive symptom group (SDS ≥40) (21). Value cuts were established based on the SDS. The SDS has been developed and proven to be quantitatively suitable for the subjective perception of depression with good validity and reliability in China, with a Cronbach's alpha coefficient and a test-retest reliability of 0.862 and 0.820, respectively.

### 2.6 Parents' educational background and family monthly income

Parents' educational background and family monthly income were also surveyed through a questionnaire.

### 2.7 Statistical methods

A Chi square test was used to determine gender differences in the SDS. We used an independent sample *t*-test to verify physical activity score, sleep quality score, and gender differences in screen time. Multiple regression analysis was performed with the depression score as the dependent variable, with gender, age, family income, education level of parents, physical activity score, sleep quality score, and screen time as predictor variables. Variables were selected according to the stepwise increase and decrease method, and we used the likelihood ratio test method to calculate and set the threshold *P*-value as 0.20. *P* < 0.05 was considered statistically significant. We used JMP 20.0J for all statistical analyses and processing (SAS Institute Inc., Cary, NC).

### 2.8 Sample size estimation

The sample size for the study was determined using the G^*^Power calculator 3.1.9.7 (Franz Faul et al., Universität Kiel, Germany, http://www.gpower.hhu.de/). Considering an α = 0.05, 1-β = 0.90, the number of tested predictors = 3 (physical activity score, screen time, and sleep quality score), the number of covariates = 3 (family income, age, and sex), we calculated the sample size to be 33, 73, and 528, respectively if the effect size f2 equaled to 0.35 (large), 0.15 (medium) and 0.02 (small). Furtherly, a 20% dropout rate was assumed, and the total number was estimated as 42–660. The actual valid sample size was 298, which was much larger than the estimated size as large effect size f2.

### 2.9 Ethics approval and consent to participate

All procedures performed in studies involving human participants were in accordance with the ethical standards of the institutional and/or national research committee and with the 1964 Helsinki Declaration and its later amendments or comparable ethical standards. All study participants provided informed consent, agreeing to the required measurement and survey completion procedures. This study was approved by the ethics committee of the Gannan Medical University, China, No: 2021110.

## 3 Results

The characteristics of the study participants are listed in Table 1. The participants' average age was 12.0 years. Their parents' education level was mostly high school and family income was mostly 3,001–5,000 yuan.

**Table 1:** Characteristics of the study subjects.

	**Mean**	**SD**	***n* (%)**
Boys (*n* = 138)			
Age	12.1	1.8	
Physical activity score	27.8	16.5	
Sleep quality score	5.3	2.1	
Screen time	101.8	3.8	
Depression score	37.9	8.0	
Girls (*n* = 158)
Age	12.0	1.7	
Physical activity score	21.6	17.0	
Sleep quality score	5.4	2.2	
Screen time	109.1	3.6	
Depression score	41.0	6.9	
Mothers (*n* = 296)			
**Education**		
Secondary school or below			120 (16)
High school			132(61)
College or above			44 (23)
Fathers (*n* = 296)			
**Education**		
Secondary school or below			104 (35)
High school			126(43)
College or above			66 (22)
Family monthly income (yuan)			
≤3,000			63 (21)
3,001–5,000			133 (45)
>5,000			100 (34)

**Figure 1:**
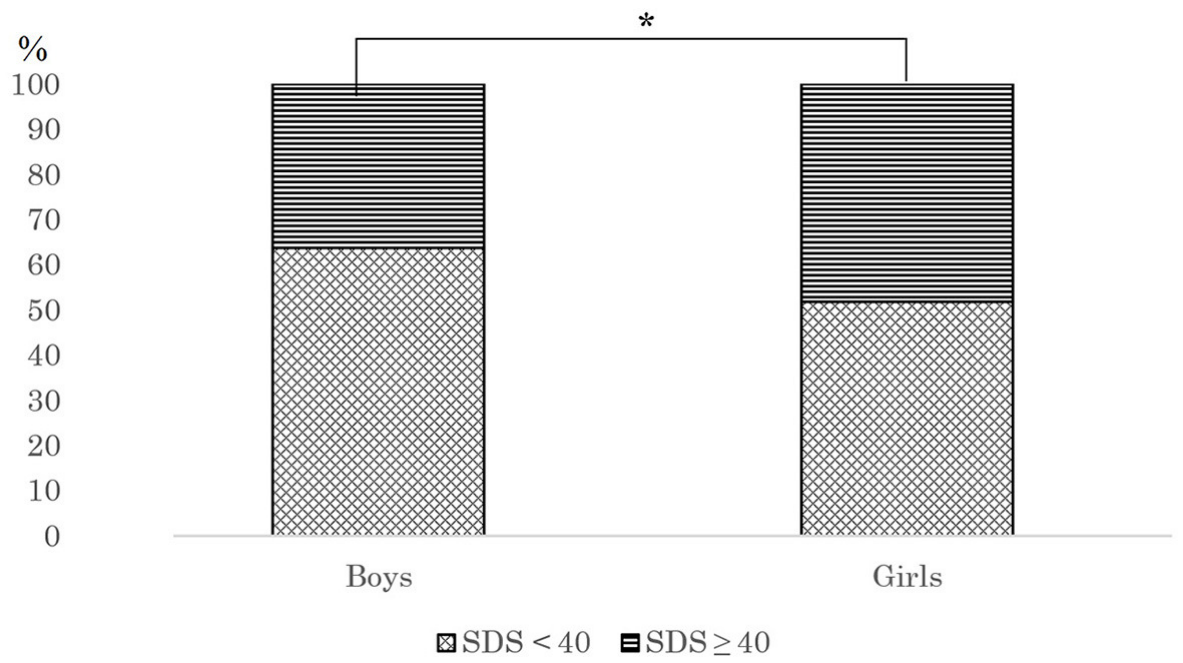
The percentage of boy and girls with SDS scores ≥40. *Chi square test, *P* < 0.05.

Table 2 shows the gender differences in lifestyle. Gender differences were found in exercise scores, but not in sleep time and screen time. In addition, we found that the proportion of females in the depression group is higher (Figure 1).

**Table 2:** Gender differences in lifestyle.

	**Mean** ±**SD**	
	**Boys (*n* = 138)**	**Girls (*n* = 158)**
Physical activity score	27.8 ± 16.5	21.6 ± 17.0^**^
Sleep quality score	5.3 ± 2.1	5.4 ± 2.2
Screen time	101.8 ± 3.8	109.1 ± 3.6

** t-test, *P* < 0.01.

Table 3 shows the results of the multiple regression analysis of the factors affecting the SDS scores. The higher the *R*-squared score, the better the representativeness of the model. R-squared for the model in this study was 0.39, *P* < 0.01. The model in this study has a certain representativeness. According to the multiple linear regression equation standardized coefficient β, the intensity of the impact on SDS scores varies from high to low, as follows: physical activity (β = −0.38, *t* = −7.06, *P* < 0.01), family income (β = −0.20, *t* = −4.07, *P* < 0.01), screen time (β = 0.16, *t* = 3.34, *P* < 0.01), age (β = 0.15, *t* = 3.16, *P* < 0.01), sex (boy: 1; girl: 0; β = −0.13, *t* = −2.74, *P* < 0.01), and sleep quality (β = −0.08, *t* = −1.87, *P* < 0.01), and these were significant predictors of the SDS scores.

**Table 3:** Factors that contributed to depression level of children (*n* = 296).

	**β**	***t***	**VIF**	***P***
Physical activity score	−0.38	−7.06	1.16	<0.01
Family income	−0.20	−4.07	1.10	<0.01
Screen time	0.16	3.34	1.09	<0.01
Age	0.15	3.16	1.10	<0.01
Sex (Boy: 1; Girl: 0)	−0.13	−2.74	1.06	<0.01
Sleep quality score	−0.08	−1.87	1.03	<0.05
Education level (Father)	0.06	1.37	1.01	>0.05

*R*^2^: 0.39; *P* < 0.01; RMES: 5.96.

## 4 Discussion

The depression levels of middle school students were similar to those of college students. A study on college students in southern China showed that the average SDS scores of male and female college students were 44.9 and 46.8, respectively (22). College students need to adapt to the increased independence and academic pressure of college life, making them prone to depression and other mental health problems (23). In China, college students have more free time to use the Internet compared to middle school students, and thus are at a higher risk of being exposed to cyberbullying behavior which can lead to depression (24). In addition, several studies have shown that impending adulthood is a period of emotional complexity during which one develops career and social orientations, enters into adult relationships, and faces potentially high levels of stress due to familial, financial, and professional obligations (25, 26) as well as frequently experiencing emotional turmoil (27). Indeed, the college years coincide with this period, thus leading to a potentially higher prevalence of depression. The findings of this study revealed that male and female middle school pupils had depression levels of 37.9 and 41.0, respectively, which is close to that of college students. However, compared to college students, middle school students have lower mental maturity and a higher possibility of extreme behavior. Studies have shown that depression may affect 2%−8% of children and adolescents; ~40% of affected children will have recurrent episodes, one-third of affected children will attempt suicide, and 3%−4% of children will die of suicide (28). It is particularly important to improve the current high depression levels among middle school students as soon as possible.

Girls had higher levels of depression than boys (Figure 1). A survey on the prevalence of depressed mood in ~12,000 Norwegian adolescents showed that girls had higher levels of depression than boys, and this difference persisted throughout adolescence (29). A screening of depressive symptoms among 4,100 adolescents in Wuhan, China, showed that the prevalence of high depressive symptoms in girls and boys was 38.9 and 30.2%, respectively, with prevalence higher for girls than boys (30). In addition, the results of a new descriptive analysis on the depression status of adolescents in Huangshi City, China, show that the prevalence of depression has been on the rise since 2018, especially in 2021, and the prevalence is higher among females than males (31). Another study on the relationship between non-educational electronic screen time and depressive symptoms among 27,070 seventh-grade middle school students in China showed that girls had a higher prevalence of depression than boys (27.6 vs. 17.7%) (32). This study supports the findings of a previous study (Figure 1). Previous studies have emphasized that physiological and psychological differences between boys and girls are important reasons for the difference in depression levels. According to epidemiological research, women are more likely than men to experience mental illnesses including anxiety and post-traumatic stress disorder, which may be associated with variations in gonadal hormone levels during stressful situations and differences in gender-specific brain regions (33). At the same time, compared with boys, girls are more susceptible to tension, more emotional, and more likely to have negative emotions, resulting in higher levels of depression (34). In addition, boys are often allowed to have more social and outdoor time, while girls are often restricted from going out and socializing (35, 36). This may further exacerbate gender differences in depression levels.

The results of this study emphasize that exercise level may be an important reason for the emergence of gender differences in depression levels among middle school students. Compared with girls, boys scored higher at the sports level (Table 2). Research has shown that physical activity can help prevent the onset and relapse of mild to moderate depression (37). In another study of the relationship between physical activity and mental disorders in U.S. adults, researchers used data from the National Comorbidity Survey (*n* = 8,089) to compare the prevalence of mental disorders among those who participated in regular physical activity and those who did not. Using multivariate logistic regression analysis, the study showed that physical activity was associated with lower levels of depression, and those who were physically active had lower levels of depression than those who did not participate in a daily exercise program (38). This study supports the findings of previous studies, confirming that higher physical activity scores are associated with lower levels of depression among middle school students in rural Northeast China (Table 3). Regular recreational exercise of any intensity could prevent future depression, according to a study of 33,908 adults in the Exercise and Depression Prevention Study (39). Therefore, it is important to reduce depression levels in middle school students, especially girls, by increasing the physical activity level of young people.

The sleep quality scores of males and females in this area were 5.3 ± 2.1 and 5.4 ± 2.2. A PSQI score >5 indicates poor sleep quality in middle school students. Sleep quality has a significant impact on mental health. Studies have shown that mental illnesses such as depression are often associated with the development of sleep problems (40). The findings of this study demonstrated a relationship between poorer sleep quality and higher levels of depression, supporting the findings of previous studies (Table 3). Studies have shown that sleep disturbance is a stand-alone risk factor for teen suicide and substance use; it is connected to depression and other psychiatric illnesses and increases the risk of suicidal behavior in depressed patients. Therefore, treating sleep disturbance in early adolescence may lower the chance of these negative results (41, 42). Data from another survey on the relationship between sleep disorders and depression in 4,175 young people aged 11–17 years showed that depression raises the likelihood of sleep loss, which in turn raises the likelihood of depression (43). Therefore, to reduce depression, it is particularly important to improve sleep in adolescents.

The results showed that the longer the use of screen time by middle school students, the higher their level of depression (Table 2). Numerous studies have demonstrated that screen time exerts a detrimental effect on depression. A study of 1,380 Egyptian college students showed that prolonged smartphone use may lead to depression (44). Another survey of 688 Lebanese university students found that mobile phone addicts were more likely to experience depression (45). Notably, previous studies have emphasized that the impact of screen time on depression results from excessive screen time encroaching on and affecting the duration and quality of sleep (46). However, the results of this study showed that compared with sleep quality, screen time has a stronger effect on depression (Table 2); even under the condition of sufficient sleep time, longer screen time itself may lead to higher depression levels. Therefore, to prevent and reduce depression levels in adolescents, screen time should be reasonably controlled even under the condition of sufficient sleep.

Findings from a previous study suggested that family income positively impacts children's emotional wellbeing (47). A study of 12,210 middle- and high-school adolescent students showed that low family income is an important factor affecting depression (48). Our results were consistent with previous findings. According to the preliminary analysis, the higher the family income of rural middle school students in Northeast China, the lower their level of depression (Table 3), which supports previous research. Although income is one of the most commonly used indicators of an individual or household's financial status, the relationship between income and depression remains unclear. Low income was not linked to depression in Spain, according to a Collaborative Research on Aging in Europe (COURAGE) study carried out in Finland, Poland, and Spain (49). Perhaps low income is not a primary risk factor associated with depression for Spaniards (50). It is possible that sunshine (51) and diet (52) play a mitigating role. Therefore, when other socioeconomic factors (such as education level and employment status) and health status are controlled for, this link is greatly reduced or even becomes insignificant (53, 54). The 2018 China Health and Retirement Longitudinal Study (CHARLS), a cross-sectional study of 16,545 individuals, showed that a higher level of economic development largely contributes to the alleviation of depression (55). A study on cognitive vulnerability to depression among Canadian and Chinese adolescents showed that Chinese adolescents had higher levels of depressive symptoms than Canadian adolescents due to differences in culture and beliefs (56). A substantial amount of research has examined the connection between socioeconomic position and mental health. There are some studies that suggest that the level of depression may vary due to cultural differences, different economic levels (55, 57), but we did not find similar results. This may be related to the concentration of regions we have chosen and the small sample size.

## 5 Limitations

Our study participants came from a single school in Benxi City. Therefore, it is difficult to generalize these results to other cities in the northeast region. As this was a cross-sectional study, we were unable to determine a causal relationship between depression levels and lifestyle habits among middle school students.

## 6 Conclusions

Among rural middle-school students in northeastern China, depression levels were much higher in girls than in boys. High sleep quality and high physical activity levels were negatively associated with depression, while low household income and high screen time were positively associated with depression. Therefore, factors such as strengthening physical activity, improving sleep quality, and reducing screen time can greatly help prevent and reduce depression.

## Data availability statement

The original contributions presented in the study are included in the article/supplementary material, further inquiries can be directed to the corresponding author.

## Ethics statement

The studies involving humans were approved by Ethics Committee of Gannan Medical University. The studies were conducted in accordance with the local legislation and institutional requirements. The participants provided their written informed consent to participate in this study.

## Author contributions

JZ: Writing—original draft, Conceptualization, Data curation, Formal analysis, Investigation, Methodology, Project administration, Resources, Software, Supervision, Validation, Visualization, Writing—review & editing. XW: Software, Writing—review & editing. SX: Software, Writing—review & editing. WY: Project administration, Writing—review & editing. JW: Validation, Writing—review & editing. EW: Software, Writing—review & editing. TL: Data curation, Writing—review & editing. MH: Conceptualization, Data curation, Formal analysis, Funding acquisition, Investigation, Methodology, Project administration, Resources, Software, Supervision, Validation, Visualization, Writing—original draft, Writing—review & editing.

## References

[1] Clarke DM Currie KC. Depression, anxiety and their relationship with chronic diseases: a review of the epidemiology, risk and treatment evidence. Med J Aust. (2009) 190:54–60. 10.5694/j.1326-5377.2009.tb02471.x 19351294

[2] Liu Q He H Yang J Feng X Zhao F Lyu J. Changes in the global burden of depression from 1990 to 2017: findings from the Global Burden of Disease study. J Psychiatr Res. (2020) 126:134–40. 10.1016/j.jpsychires.2019.08.002 31439359

[3] Mathers CD Loncar D. Projections of global mortality and burden of disease from 2002 to 2030. PLoS Med. (2006) 3:e442. 10.1371/journal.pmed.0030442 17132052 PMC1664601

[4] Shorey S Ng ED Wong CHJ. Global prevalence of depression and elevated depressive symptoms among adolescents: a systematic review and meta-analysis. Br J Clin Psychol. (2021) 61:287–305. 10.1111/bjc.12333 34569066

[5] Liu C Pan W Zhu D Meng F Tian T Li L. Factors of suicidal behavior among inpatients with major depressive disorder: a retrospective case series. Front Psychiatry. (2022) 13:996402. 10.3389/fpsyt.2022.996402 36213915 PMC9537680

[6] Cipriani A Barbui C Butler R Hatcher S Geddes J. Depression in adults: drug and physical treatments. BMJ Clin Evid. (2011) 2011:1003. PMC3217759 21609510

[7] Taheri M. Comparative study of the long-term impact of the COVID-19 pandemic on mental health and nutritional practices among international elite and sub-elite athletes: a sample of 1420 participants from 14 countries. Sports Med-Open. (2023) 9:104. 10.1186/s40798-023-00653-w 37938473 PMC10632320

[8] Pranoto NW. The effects of inactivity during the COVID-19 pandemic on the psychomotor skills of kindergarten students. Ann Appl Sport Sci. (2023). 10.52547/aassjournal.1162

[9] Afshar-Zanjani H Khalifesoltani F Hajializadeh K Ahadi H. Comparison of the effectiveness of self-efficacy-based training and compassion-focused therapy on depression, self-care behaviors, and quality of life of patients with irritable bowel syndrome. Int J Body Mind Cult. (2021) 8. 10.22122/ijbmc.v8i1.287

[10] Choi KW Chen CY Stein MB Klimentidis YC Wang MJ Koenen KC. Assessment of bidirectional relationships between physical activity and depression among adults: a 2-sample Mendelian Randomization Study. JAMA Psychiatry. (2019) 76:399–408. 10.1001/jamapsychiatry.2018.4175 30673066 PMC6450288

[11] Zhang CS Cheng L Chen X Wang Y Wei S Sun J. The strategies of exercise intervention for adolescent depression: a meta-analysis of randomized controlled trials. Front Psychol. (2022) 13:974382. 10.3389/fpsyg.2022.974382 36687827 PMC9846179

[12] Baiden P Spoor SP Nicholas JK Brown FA. LaBrenz, CA, Spadola, C. Association between use of electronic vaping products and insufficient sleep among adolescents: findings from the 2017 and 2019 YRBS. Sleep Med. (2023) 101:19–27. 10.1016/j.sleep.2022.10.005 36334497

[13] Mohan J Xiaofan G Yingxian S. Association between sleep time and depression: a cross-sectional study from countries in rural Northeastern China. J Int Med Res. (2017) 45:984–92. 10.1177/0300060517701034 28425823 PMC5536416

[14] Lee JE Jang SI Ju YJ Kim W Lee HJ Park EC. Relationship between mobile phone addiction and the incidence of poor and short sleep among Korean Adolescents: a Longitudinal Study of the Korean Children and Youth Panel Survey. J Korean Med Sci. (2017) 32:1166–72. 10.3346/jkms.2017.32.7.1166 28581275 PMC5461322

[15] Ng KC Wu LH Lam HY Lam LK Nip PY Ng CM. The relationships between mobile phone use and depressive symptoms, bodily pain, and daytime sleepiness in Hong Kong secondary school students. Addict Behav. (2020) 101:105975. 10.1016/j.addbeh.2019.04.033 31076240

[16] Hankin BL Abramson LY Moffitt TE Silva PA McGee R Angell KE. Development of depression from preadolescence to young adulthood: emerging gender differences in a 10-year longitudinal study. J Abnorm Psychol. (1998) 107:128–40. 10.1037//0021-843X.107.1.128 9505045

[17] Qu M Yang K Ren H Wen L Tan S Xiu M. The impact of school education on depressive symptoms in Chinese adolescents: a prospective longitudinal study. Int J Ment Health Addict. (2022) 1–15. 10.1007/s11469-022-00944-5 36406902 PMC9651092

[18] Li C Liang Z Yin X Zhang Q. Family social capital mediates the effect of poverty on children's anxiety and depression. J Community Psychol. (2018) 46:983–95. 10.1002/jcop.22086 30311971

[19] Yang G Li Y Liu S. Physical activity influences the mobile phone addiction among Chinese undergraduates: the moderating effect of exercise type. J Behav Addict. (2021) 10:799–810. 10.1556/2006.2021.00059 34546969 PMC8997213

[20] Buysse DJ Reynolds CF Monk TH Berman SR Kupfer DJ. The Pittsburgh Sleep Quality Index: a new instrument for psychiatric practice and research. Psychiatry Res. (1989) 28:193–213. 10.1016/0165-1781(89)90047-4 2748771

[21] Zung WWA. self-rating depression scale. Arch Gen Psychiatry. (1965) 12:63–70. 10.1001/archpsyc.1965.01720310065008 14221692

[22] Wu C Hao M Liu X Yang D Liu B Yan W. The effects of body dissatisfaction and depression levels on the dietary habits of university students in southern China during COVID-19. Front Nutr. (2023) 10:1103724. 10.3389/fnut.2023.1103724 37599684 PMC10434794

[23] Hao M Liu X Wang Y Wu Q Yan W Hao Y. The associations between body dissatisfaction, exercise intensity, sleep quality, and depression in university students in southern China. Front Psychiatry. (2023) 14:1118855. 10.3389/fpsyt.2023.1118855 37020733 PMC10067572

[24] Wang W Xie X Wang X. Cyberbullying and depression among Chinese college students: a moderated mediation model of social anxiety and neuroticism. J Affect Disord. (2019) 256:54–61. 10.1016/j.jad.2019.05.061 31158716

[25] Huang Y Heflin CM Validova A. Material hardship, perceived stress, and health in early adulthood. Ann Epidemiol. (2021) 53:69–75.e3. 10.1016/j.annepidem.2020.08.017 32949721 PMC7494502

[26] Fenzel LM Richardson KD. The stress process among emerging adults: spirituality, mindfulness, resilience, and self-compassion as predictors of life satisfaction and depressive symptoms. J Adult Dev. (2022) 29:1–15. 10.1007/s10804-021-09384-2

[27] Feldt R Bejar M Lee J Louison R. Vocational identity resources in emerging adulthood: associations with facets of dispositional mindfulness. Career Dev Q. (2021) 69:2–18. 10.1002/cdq.12245

[28] Hazell P. Depression in children and adolescents. BMJ Clin Evid. (2011) 2011:1008. 22018419

[29] Wichstrøm L. The emergence of gender difference in depressed mood during adolescence: the role of intensified gender socialization. Dev Psychol. (1999) 35:232–45. 10.1037/0012-1649.35.1.232 9923478

[30] Sun W Mei J Wang Y Zhao X Zhu Z Zhang C. Psycho-social factors associated with high depressive symptomatology in female adolescents and gender difference in adolescent depression: an epidemiological survey in China's Hubei Province. BMC Psychiatry. (2021) 21:168. 10.1186/s12888-021-03165-7 33771118 PMC7995784

[31] Zhang X Yan Y Ye Z Xie J. Descriptive analysis of depression among adolescents in Huangshi, China. BMC Psychiatry. (2023) 23:176. 10.1186/s12888-023-04682-3 36927404 PMC10019414

[32] Wang H Bragg F Guan Y Zhong J Li N Pan J. Association between duration of electronic screen use for non-educational purposes and depression symptoms among middle and high school students: a cross-sectional study in Zhejiang Province, China. Front Public Health. (2023) 11:1138152. 10.3389/fpubh.2023.1138152 37261230 PMC10229063

[33] Maeng LY Milad MR. Sex differences in anxiety disorders: interactions between fear, stress, and gonadal hormones. Horm Behav. (2015) 76:106–17. 10.1016/j.yhbeh.2015.04.002 25888456 PMC4823998

[34] Hankin BL Abramson LY. Development of gender differences in depression: description and possible explanations. Ann Med. (1999) 31:372–9. 10.3109/07853899908998794 10680851

[35] Ninsiima AB Leye E Michielsen K. “Girls have more challenges; they need to be locked up”: a qualitative study of gender norms and the sexuality of young adolescents in Uganda. Int J Environ Res Public Health. (2018) 15:193. 10.3390/ijerph15020193 29364192 PMC5858264

[36] Fang P Sun L Shi SS. Influencing factors related to female sports participation under the implementation of chinese government interventions: an analysis based on the China family panel studies. Front Public Health. (2022) 10:875373. 10.3389/fpubh.2022.875373 35719610 PMC9201213

[37] Andersson E Hovland A Kjellman B Taube J Martinsen E. Physical activity is just as good as CBT or drugs for depression. Lakartidningen. (2015) 112:DP4E. 26574804

[38] Goodwin RD. Association between physical activity and mental disorders among adults in the United States. Prev Med. (2003) 36:698–703. 10.1016/S0091-7435(03)00042-2 12744913

[39] Harvey SB Øverland S Hatch SL Wessely S Mykletun A Hotopf M. Exercise and the prevention of depression: results of the HUNT Cohort Study. Am J Psychiatry. (2018) 175:28–36. 10.1176/appi.ajp.2017.16111223 28969440

[40] Nunes ML Bruni O. Insomnia in childhood and adolescence: clinical aspects, diagnosis, and therapeutic approach. J Pediatr. (2015) 91:26–35. 10.1016/j.jped.2015.08.006 26392218

[41] de Zambotti M Goldstone A Colrain IM Baker FC. Insomnia disorder in adolescence: diagnosis, impact, and treatment. Sleep Med Rev. (2018) 39:12–24. 10.1016/j.smrv.2017.06.009 28974427 PMC5931364

[42] Wang X Cheng S Xu H. Systematic review and meta-analysis of the relationship between sleep disorders and suicidal behaviour in patients with depression. BMC Psychiatry. (2019) 19:303. 10.1186/s12888-019-2302-5 31623600 PMC6798511

[43] Roberts RE Duong HT. The prospective association between sleep deprivation and depression among adolescents. Sleep. (2014) 37:239–44. 10.5665/sleep.3388 24497652 PMC3900610

[44] Okasha T Saad A Ibrahim I Elhabiby M Khalil S Morsy M. Prevalence of smartphone addiction and its correlates in a sample of Egyptian university students. Int J Soc Psychiatry. (2021) 68:1580–8. 10.1177/00207640211042917 34479450

[45] Matar Boumosleh J Jaalouk D. Depression, anxiety, and smartphone addiction in university students- a cross sectional study. PLoS ONE. (2017) 12:e0182239. 10.1371/journal.pone.0182239 28777828 PMC5544206

[46] Wang W Du X Guo Y Li W Zhang S Zhang W. Associations among screen time, sleep duration and depressive symptoms among Chinese adolescents. J Affect Disord. (2021) 284:69–74. 10.1016/j.jad.2021.01.082 33582434

[47] Qi D Wu Y. Family income and children's emotional wellbeing: the mediational role of parents' life satisfaction and emotional wellbeing in China. Int J Environ Res Public Health. (2020) 17:7573. 10.3390/ijerph17207573 33080999 PMC7589884

[48] Lin HC Tang TC Yen JY Ko CH Huang CF Liu SC. Depression and its association with self-esteem, family, peer and school factors in a population of 9586 adolescents in southern Taiwan. Psychiatry Clin Neurosci. (2008) 62:412–20. 10.1111/j.1440-1819.2008.01820.x 18778438

[49] Freeman A Tyrovolas S Koyanagi A Chatterji S Leonardi M Ayuso-Mateos JL. The role of socio-economic status in depression: results from the COURAGE (aging survey in Europe). BMC Public Health. (2016) 16:1098. 10.1186/s12889-016-3638-0 27760538 PMC5069819

[50] Tuesca-Molina R Fierro Herrera N Molinares Sosa A Oviedo Martínez F Polo Arjona Y Polo Cueto J. Socializing groups as protective factor against depression in elderly people: Barranquilla, Colombia. Rev Esp Salud Publica. (2003) 77:595–604. 10.1590/S1135-57272003000500008 14608962

[51] Kent ST McClure LA Crosson WL Arnett DK Wadley VG Sathiakumar N. Effect of sunlight exposure on cognitive function among depressed and non-depressed participants: a REGARDS cross-sectional study. Environ Health. (2009) 8:34. 10.1186/1476-069X-8-34 19638195 PMC2728098

[52] Virtanen M Koskinen S Kivimäki M Honkonen T Vahtera J Ahola K. Contribution of non-work and work-related risk factors to the association between income and mental disorders in a working population: the Health 2000 Study. Occup Environ Med. (2008) 65:171–8. 10.1136/oem.2007.033159 18283127

[53] Skarupski KA Tangney CC Li H Evans DA Morris MC. Mediterranean diet and depressive symptoms among older adults over time. J Nutr Health Aging. (2013) 17:441–5. 10.1007/s12603-012-0437-x 23636545 PMC4454450

[54] Martikainen P Adda J Ferrie JE Davey Smith G Marmot M. Effects of income and wealth on GHQ depression and poor self rated health in white collar women and men in the Whitehall II study. J Epidemiol Community Health. (2003) 57:718–23. 10.1136/jech.57.9.718 12933779 PMC1732572

[55] Wang J Zhang J Lin H Han Y Tu J Nie X. Economic development, weak ties, and depression: evidence from China. J Affect Disord. (2023) 334:246–57. 10.1016/j.jad.2023.04.097 37146909

[56] Auerbach RP Eberhart NK Abela JR. Cognitive vulnerability to depression in Canadian and Chinese adolescents. J Abnorm Child Psychol. (2010) 38:57–68. 10.1007/s10802-009-9344-y 19669872 PMC2809945

[57] Aldwin C Greenberger E. Cultural differences in the predictors of depression. Am J Community Psychol. (1987) 15:789–813. 10.1007/BF00919803 3439551

